# Comprehensive review of additively manufactured biodegradable magnesium implants for repairing bone defects from biomechanical and biodegradable perspectives

**DOI:** 10.3389/fchem.2022.1066103

**Published:** 2022-11-29

**Authors:** Zhengguang Wang, Bingchuan Liu, Bangzhao Yin, Yufeng Zheng, Yun Tian, Peng Wen

**Affiliations:** ^1^ Department of Orthopaedics, Peking University Third Hospital, Beijing, China; ^2^ Engineering Research Center of Bone and Joint Precision Medicine, Ministry of Education, Peking University Third Hospital, Beijing, China; ^3^ Department of Mechanical Engineering, Tsinghua University, Beijing, China; ^4^ School of Materials Science and Engineering, Peking University, Beijing, China

**Keywords:** bibilometric analysis, magnesium–alloy, additive manucatruing, biomechanical properties, biodegradation behaviours

## Abstract

Bone defect repair is a complicated clinical problem, particularly when the defect is relatively large and the bone is unable to repair itself. Magnesium and its alloys have been introduced as versatile biomaterials to repair bone defects because of their excellent biocompatibility, osteoconductivity, bone-mimicking biomechanical features, and non-toxic and biodegradable properties. Therefore, magnesium alloys have become a popular research topic in the field of implants to treat critical bone defects. This review explores the popular Mg alloy research topics in the field of bone defects. Bibliometric analyses demonstrate that the degradation control and mechanical properties of Mg alloys are the main research focus for the treatment of bone defects. Furthermore, the additive manufacturing (AM) of Mg alloys is a promising approach for treating bone defects using implants with customized structures and functions. This work reviews the state of research on AM-Mg alloys and the current challenges in the field, mainly from the two aspects of controlling the degradation rate and the fabrication of excellent mechanical properties. First, the advantages, current progress, and challenges of the AM of Mg alloys for further application are discussed. The main mechanisms that lead to the rapid degradation of AM-Mg are then highlighted. Next, the typical methods and processing parameters of laser powder bed fusion fabrication on the degradation characteristics of Mg alloys are reviewed. The following section discusses how the above factors affect the mechanical properties of AM-Mg and the recent research progress. Finally, the current status of research on AM-Mg for bone defects is summarized, and some research directions for AM-Mg to drive the application of clinical orthopedic implants are suggested.

## 1 Introduction

Treatment and repair of large bone-defects is a global problem in orthopedics because when the defect site exceeds a critical size limit, the bone is often unable to heal spontaneously. Trauma, infection, tumors, and other factors can cause irreversible bone defects (Ganguly et al., 2022). More than two million bone grafting operations are performed annually (Zhang et al., 2019). Currently, autologous bone, artificial bone, allogeneic bone graft, and bone-handling methods cannot meet clinical needs. Traditional metallic biomaterials for bone defect implantation are mainly based on inert metals such as Ti. Although these materials offer excellent corrosion resistance and mechanical loading properties, their drawback is the mismatch between their mechanical properties, especially their elastic modulus and tensile strength, which are significantly higher than those of human bones (Li et al., 2019a). Therefore, the stress shielding phenomenon is inevitable, suppressing bone regeneration and resulting in implant inflammation and loosening (Bose et al., 2021).

When treating bone defects, these inert metal implants permanently remain in the body after implantation and must be removed after healing. Therefore, the development of biodegradable implants that are resorbable and disappear from the body after bone reunion has attracted research interest. Magnesium-based alloys are promising next-generation implant biomaterials owing to their outstanding biocompatibility, biodegradability, and mechanical compatibility as temporary *in vivo* bone substitutes. Apart from material selection, the size and shape of a fixation implant should ideally be tailored to a specific bone defect situation, with a microstructure mimicking that of the host bone. With the advent of sophisticated laser systems and platforms, additive manufacturing (AM) has become ideal for addressing these issues. However, Mg-based alloys are susceptible to accelerated and localized corrosion, which could result in the unintended early failure of the implant. In this review, the progress in the AM of biodegradable Mg-based alloys for bone repair applications is reviewed. Laser powder bed fusion (L-PBF) is the predominant AM technique used for the fabrication of AM-Mg alloys. The impacts of the powder properties and AM processing parameters on the biomechanical and biodegradation properties are discussed and outlined. The mechanical properties and corrosion resistance of L-PBF-fabricated Mg-based alloys are summarized.

### 1.1 Biodegradable characteristics of Mg alloys as bone implants

Magnesium is essential for supporting and sustaining health and life. It is closely related to the stability and formation of the bone environment, has good biosafety and biological activity, and promotes bone regeneration (de Baaij et al., 2015). Table 1 summarizes the unique advantages and drawbacks of Mg as a versatile bone repair material in biomechanics and biomedicine. Magnesium ion (Mg^2+^) is essential for bone homeostasis and metabolism, and its deficiency disrupts systemic bone metabolism, leading to insufficient bone formation and uncontrolled bone resorption (Qiao et al., 2021). Additionally, Mg is an essential mineral that dissolves in body fluids or plasma, and if the degradation rate of Mg is controlled, no residue will remain after healing is complete. The recommended daily intake amount of Mg is approximately 300–400 mg, and excess Mg ions are effectively excreted from the body through urine (Liu et al., 2019a). Hence, surgical reoperations for implant removal can be avoided.

**TABLE 1:** Biomechanical and biomedical aspects illustrating the key advantages and disadvantages of AM Mg alloys as orthopedic implants (Radha and Sreekanth, 2017).

Characteristics	Details
Biomechanical benefits	
Significant similarities with human bone	The density of Mg (1.738 g/cm^3^) is very similar to that of the cortical bone (1.75–2.1 g/cm^3^)
Bone-mimicking mechanical properties	The Mg matrix possesses mechanical properties similar to that of bone tissue, high-specific strength, and high-specific stiffness
High processibility and dimensional accuracy	Magnesium is the most practical structural metal to process and fine-tune, making it easy to achieve consistent final dimensions. Consequently, advanced models are readily producible, which is critical for the complex shapes often required for medical applications
Reduced stress shielding	As the modulus of elasticity of Mg (40–45 GPa) is more similar to that of natural bone (3–20 GPa), stress-shielding issues can be significantly avoided
Non-toxic degradation	The corrosion of Mg in the body will eventually lead to complete decomposition, avoiding post-healing surgical implant removal. The corrosion of Mg results in a non-toxic oxide in the body that can be expelled through the urine
Biomechanical Drawbacks	
Rapid degradation	A fully biocompatible Mg alloy is necessary to prevent harmful alloying elements and provide an adequate release rate for other features, including those naturally occurring
High H evolution	The emitted H_2_ gas accumulates at a rapid pace in the surrounding soft tissues. Hydrogen bubbles may compromise wound healing and cause tissue necrosis at the site of implantation
Biomedical Benefits	
Osteogenic differentiation	Magnesium enhances osteoblast differentiation by increasing collagen-X secretion and activating transcription factor 4 (ATF4) and β-catenin (β-Cat), promoting bone formation by the upregulation of Wnt/β-catenin signaling (Xu et al., 2022)
Anti-bacterial effect in the early phase	The release of Mg into the bone microenvironment can decrease biofilm formation and prevent methicillin-resistant *Staphylococcus aureus* -induced implant-related infection by promoting M1 macrophage polarization (Zheng et al., 2020)
Angiogenesis enhancement	Magnesium maintains the “vascular-bone” coupling angiogenesis by stimulating osteoblasts to secrete vascular endothelial growth factor (VEGF) and promoting the formation of H-type blood vessels (Wang et al., 2016; Gao et al., 2020)
Anti-carcinogenesis effect	Exerts photothermal effect for tumor, such as osteosarcoma, eradication (Long et al., 2021)
Enhancing peripheral nerve regeneration	Mg^2+^ promotes peripheral nerve outgrowth in a concentration-dependent trend by upregulating the PI3K/Akt pathway and Sema5b (Yao et al., 2022)
Biomedical Drawbacks	Details
Inflammatory response	The degradation debris can cause an inflammatory response in the body
Alkalosis	Owing to rapid degradation, local alkalinization around the Mg scaffold with an increase in OH^−^ ions can lead to alkalosis of the surrounding tissue when the pH > 7.8

**FIGURE 1:**
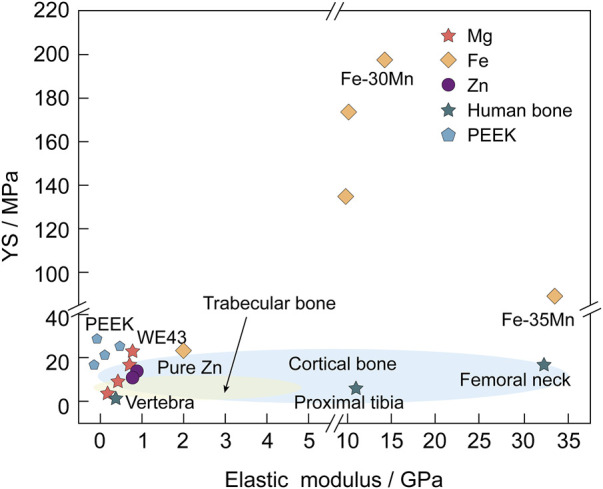
YS (MPa) and elastic modulus (GPa) of Mg and its alloys are more similar to natural bone than those of other biodegradable metallic materials (such as Fe and Zn) and Polyetheretherketone (PEEK) (Chen et al., 2022; Wang et al., 2022).

Simultaneously, Mg alloys, as compared to other biomaterials, possess a density and elastic modulus similar to that of natural bone, thus minimizing the stress shielding effect and stimulating the fractured part of the bone, promoting osteoblast differentiation and preventing osteoporosis (Figure 1). Moreover, Mg alloys are more flexible than ceramics and exhibit higher strength than polymeric materials. However, some inherent properties of Mg alloys, such as rapid degradation and poor biomechanical properties, restrict their large-scale application.

### 1.2 Bibliometric analysis of the popular research topics on Mg alloys for bone defects

The relevant literature until 20 July 2022 was systematically retrieved from the Web of Science core collection according to the subject term retrieval of “Mg alloys and bone defects or bone repair.” There were 323 documents, including reviews and articles. Then, the inclusion criteria were established, which included 1) a primary focus on Mg alloy research and 2) a focus on the discussion of bone defects or bone repair applications, and the papers were screened accordingly.

**FIGURE 2:**
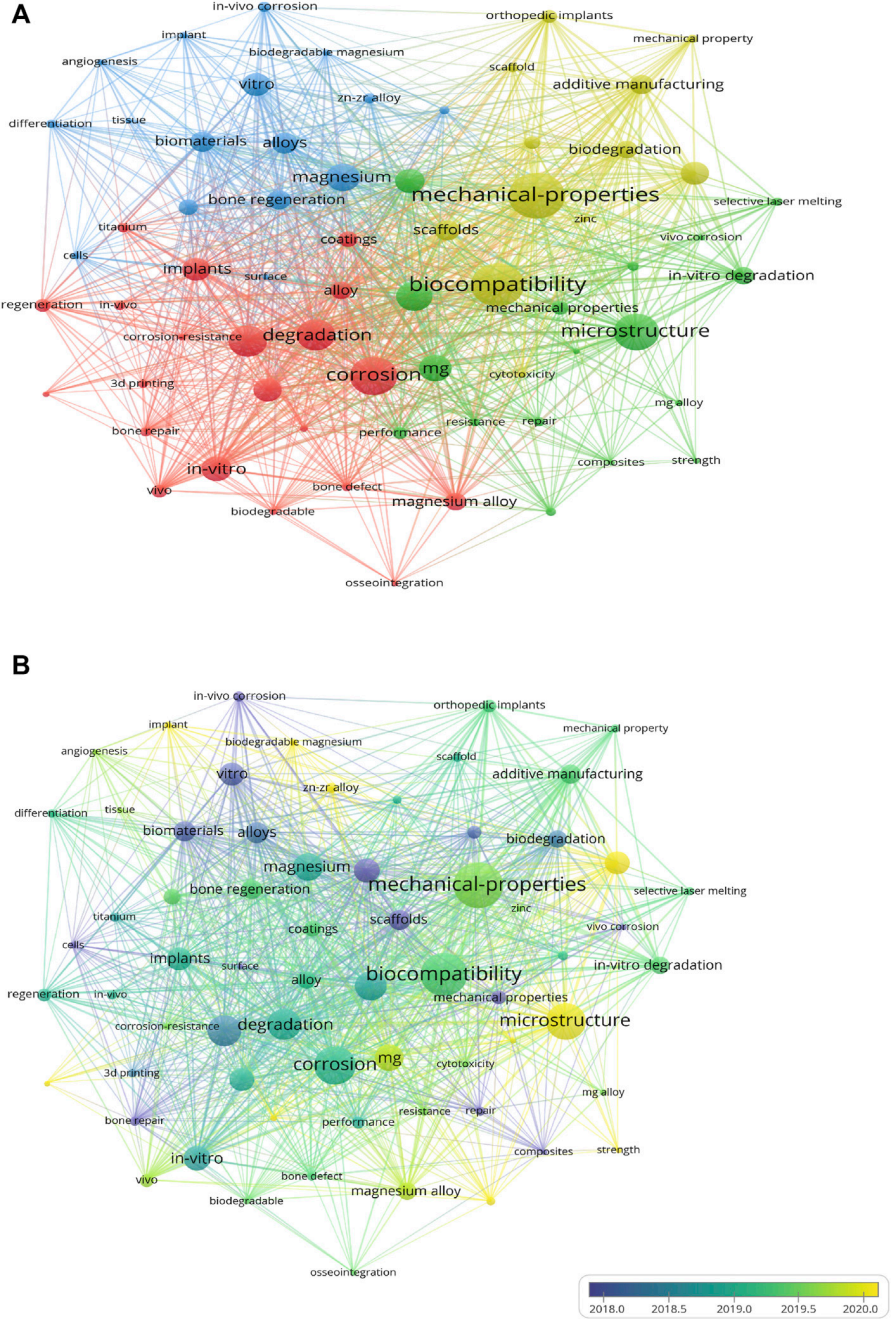
Co-occurrence analysis of the global research on Mg alloys and bone defects in the Web of Science **(A)** Co-occurrence analysis of keywords on Mg alloys and bone repair **(B)** The distribution of keyword contributions is based on the average frequency of occurrence. Blue and green keywords emerge earlier than yellow ones.

Highly-cited keywords reflect the popular research topics and issues within a discipline. Utilizing VOSviewer, keywords were extracted from the titles and abstracts of 141 eligible publications. A total of 128 keywords with more than 15 co-occurrences were subjected to mapping analysis, which yielded four clusters, namely, Cluster 1 (corrosion-resistance research, red), Cluster 2 (biomechanical research, green), Cluster 3 (Mg research, blue), and Cluster 4 (AM research, yellow), as depicted in Figure 2A. The size of each keyword was proportional to its occurrence frequency. In Cluster 1, the relevant keywords included degradation (171 times), corrosion (188 times), corrosion resistance (71 times), alloy (194 times), and coatings (62 times). In Cluster 2, the frequently appearing keywords were microstructure (186 times), *in vitro* degradation (95 times), and selective laser melting (SLM) (44 times). In Cluster 3, the main keywords were Mg (133 times), biomaterials (114 times), *in vitro* (122 times), and bone regeneration (83 times). In Cluster 4, the main keywords were mechanical properties (241 times), AM (108 times), biocompatibility (247 times), and orthopedic implants (84 times) (Supplementary Table S1).

The recent popular topics are “mechanical properties” and “microstructure” (Cluster 4). Research on mechanical properties is popular in the “biomechanical research” and “AM” clusters. The microstructure is also an essential component of the “biomechanical research” cluster. Furthermore, microstructure refinement is a method used to enhance the mechanical properties of alloys (Xu et al., 2021a), indicating that the effect of adjusting the microstructure of Mg alloys to improve their biomechanical properties has gained increasing interest. In the “AM” cluster, AM is closely related to biomechanics and degradation properties, which indicates that regulating the degradation rate of Mg biomaterials and biomechanical properties are the two most critical research topics in the field of AM Mg alloys. Notably, the keyword “orthopedic implant” appears in the “AM” and “corrosion research” clusters, indicating that AM and the modulation of the corrosion rate of Mg alloys are closely connected to clinical orthopedic applications. In the “biomechanical research” cluster, a relatively novel keyword is L-PBF, which is a technique widely applied in the AM of Mg alloys, indicating that the relationship between L-PBF and biomechanical properties has become a relatively novel popular research topic (Ahmadi et al., 2022). As shown in Figure 2B, the “biomechanical research,” “corrosion-resistance research,” and “AM research” clusters have received significantly more attention in recent years than the “Mg research” cluster. This phenomenon indicates that research on Mg alloys in bone defects has shifted from the biomedical properties of Mg to the biomechanical and corrosion properties of AM Mg alloys.

Overall, AM-based Mg fabrication improvements and microstructural modifications have become more prominent in this field by exploring the current status and research timeline of Mg alloys in bone defects. The following section reviews the progress of research on improving the biomechanical and degradation performance of AM Mg alloys.

## 2 AM and Mg alloys for bone repair

### 2.1 The combination of AM fabrication and biodegradable Mg-based alloys for treating bone defects

**FIGURE 3:**
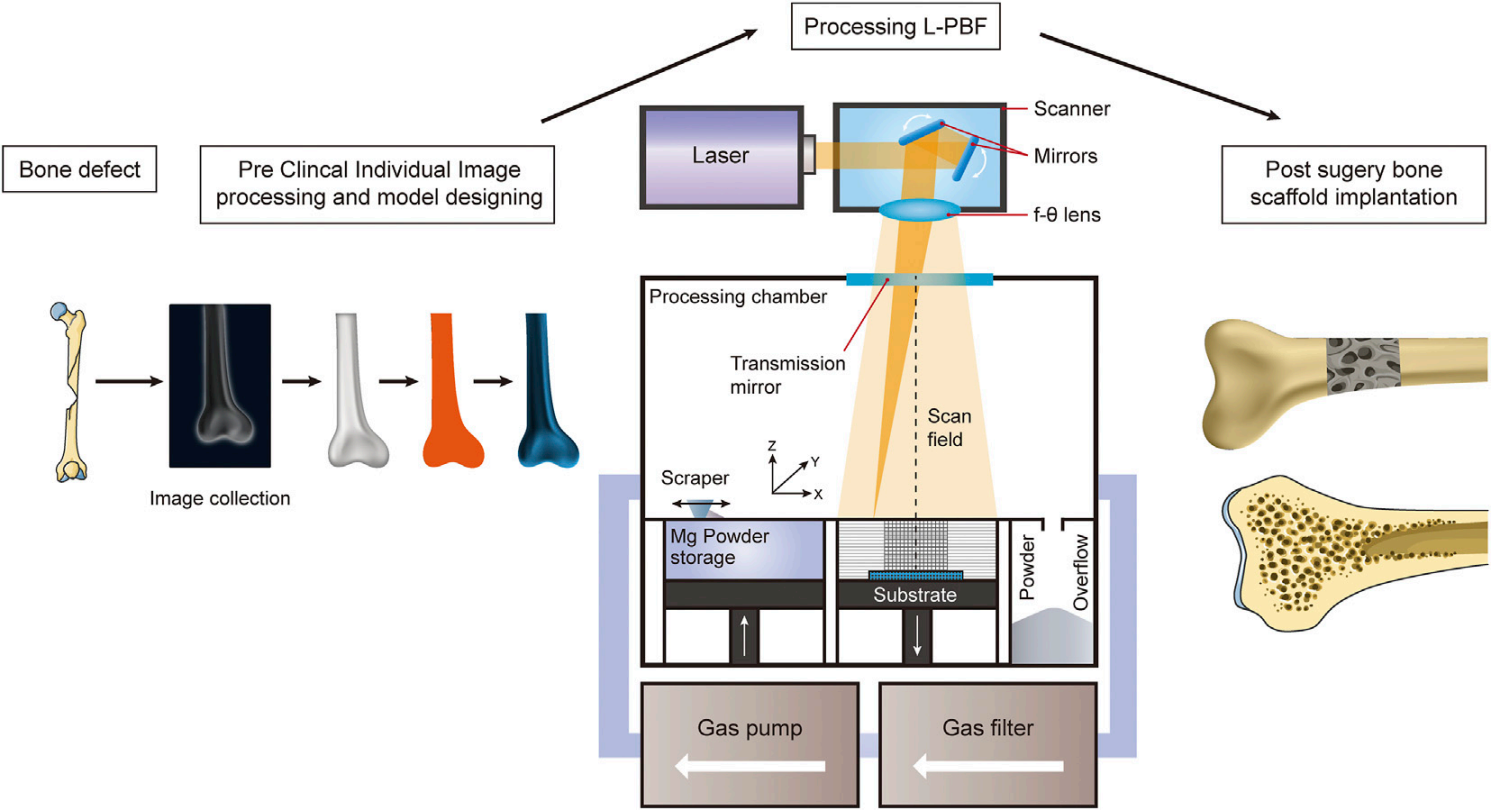
Schematic diagram illustrating the AM of Mg-alloys for the treatment of bone defects using the L-PBF technique.

Additive manufacturing, which is three-dimensional (3D) printing, is a type of manufacturing technology that builds materials into objects by sequentially building them layer-by-layer from 3D computer-aided design data. It provides a reliable way to obtain personalized and complex 3D structures and efficiently and reliably replicate anatomical forms (Murphy et al., 2020). ASTM F2792-12a (Izdebska-Podsiadły and Izdebska-Podsiadły, 2022), published in 2012, classifies AM technologies into seven categories, namely, vat polymerization, material jetting, material extrusion, powder bed fusion (PBF), binder jetting, sheet lamination, and direct energy deposition (DED). DED and PBF, in particular, are the two main processes for direct AM metal printing (DebRoy et al., 2018). DED mainly fabricates rough-shaped components and PBF is considered suitable for building complex porous implants (DebRoy et al., 2018). PBF, as compared to DED, provides lower spot diameters, finer powders, and thinner forming layers (Qin et al., 2019). SLM, also known as L-PBF, facilitating reliable stress transfer, and is advantageous for the preparation of alloys with fine microstructures and homogeneous chemical compositions (Figure 3) (Qin et al., 2019).

The application of AM to Ti scaffolds for treating bone defects is currently being tested clinically. Some progress has been made, as compared to approaches that require allogeneic/autogenous bone fillers (Hou et al., 2020; Zhang et al., 2021a). However, Ti alloys have a high risk of causing the body to reject implants by triggering an innate immune response and complications such as bone non-healing due to stress shielding. The combination of AM fabrication and biodegradable Mg-based alloys is expected to solve the current dual challenges in the treatment of bone defects, namely the two main problems of “traditional medical inert implants requiring secondary surgical removal” and “the fabrication of personalized porous implants with high precision and complex structures.”

The leading causes of bone defects include trauma, osteomyelitis, and postoperative bone tumor resection. Large bone defects due to these causes urgently require an ideal personalized bone graft to achieve mechanical stability. Several experts have already achieved breakthroughs in the treatment of osteosarcoma (Long et al., 2021), promotion of bone implant healing (Golafshan et al., 2020; Zhang et al., 2022), and inhibition of implant infections (Xie et al., 2022) using AM combined with Mg alloys. These different application scenarios demonstrate that AM-Mg will play an increasingly important role in the treatment of bone defects caused by different etiologies. These innovative studies provide a basis for the functionality of AM-Mg in the treatment of bone defects. However, the processing parameters are diverse because of the different AM techniques used. Choosing an appropriate AM method is a prerequisite for ensuring the effectiveness of AM-Mg. Therefore, ensuring that all fabricated AM-Mg implants can provide adequate mechanical support to patients in all phases of implantation and that they match the fracture healing cycle poses a new challenge for researchers.

### 2.2 L-PBF as the mainstream manufacturing technology for AM Mg alloys

**FIGURE 4:**
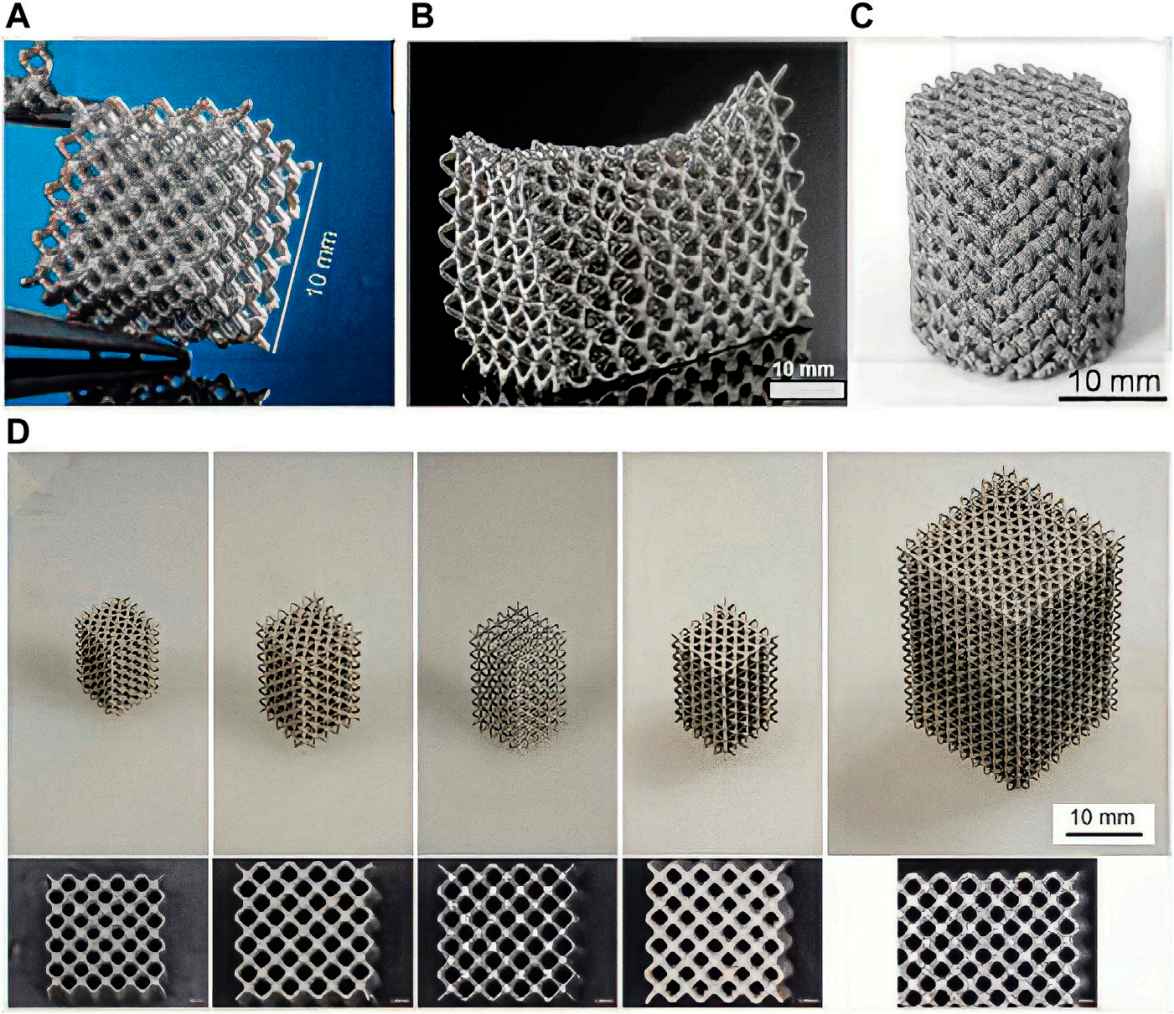
LAM WE43 scaffolds with different structural designs. **(A–D)** WE43 metal scaffolds made by L-PBF process.

L-PBF is a laser AM technology based on the layered construction of complex customized implants using high-intensity laser beams. Therefore, it has been extensively investigated for the fabrication of bone implants using polymers (Kamboj et al., 2021), metals (Xia et al., 2023a), and their composites (Liu et al., 2022a) for bone tissue engineering. L-PBF has shown excellent performance in customizing products with complex shapes. The L-PBF process is characterized by high laser energy, short printing time, and fast cooling rate, reaching more than 10^5^ K/s. Therefore, this technology has unparalleled superiority for manufacturing complex Mg alloy components with enhanced microstructures and biomechanical properties (Xu et al., 2019). The L-PBF process involves rapid heating and cooling cycles, which inhibit grain growth by rapid solidification at high cooling rates (Zeng et al., 2022). L-PBF also reduces composition segregation and allows the formation of homogeneous microstructures through the scaffolds. Figure 3 shows a schematic of the fabrication process of AM Mg porous implants using L-PBF. A dense and homogeneous microstructure favors densification, mechanical properties, and resistance to degradation in terms of phase morphology and grain size (Figures 4A–D) (Narita et al., 2019).

During fabrication, a significant portion of the laser energy is reflected by the low-energy absorption rate of Mg. Method improvements should be made to ensure that most of the energy is transferred to the material for absorption. Another notable consideration is that a continuous gas flow should carefully remove the evaporated fumes emitted during the L-PBF of Mg. Furthermore, special care should be taken to prevent the continuous flow of shielding gas from interfering with lightweight Mg powder beds (Qin et al., 2019).

### 2.3 Challenges in the AM of biodegradable Mg

Biodegradable AM metals, particularly Mg, as compared to bioinert AM Ti or Co-Cr, present new fabrication difficulties when using L-PBF processes. Intense evaporation and high chemical reactivity remain significant obstacles in the AM of Mg-based alloy powders (Gangireddy et al., 2019). First, the high affinity of Mg for O contributes to the flammability and explosiveness of Mg powder during printing (Li et al., 2018a). The Mg dust condensed from the Mg vapor formed during the L-PBF process further increases the surface area and is more prone to exothermic solid reactions and severe dust explosions. Second, evaporation during the printing process is a serious problem. The Mg matrix is melted by the laser beam, causing violent evaporation. This laser fabrication method changes the chemical makeup of the formed sample and causes flaws, such as keyholes, to form. Third, because Mg has a high thermal expansion, good wettability, and high surface tension, Mg alloys fabricated using L-PBF are susceptible to thermal deformation and powder adhesion (Carluccio et al., 2020). A poor formation quality leads to unstable properties. Regarding the degradation rate of Mg alloys, the refinement of Mg grains due to the rapid cooling and processing of L-PBF leads to increased grain boundaries and faster corrosion rates (Gollapudi, 2012).

## 3 Degradation behavior and typical strategies to enhance the corrosion protection of AM-Mg alloys

### 3.1 The current challenge of the fast degradation rate of Mg alloys

Approximately three to 4 months are required for the fracture ends of bone-degradable metallic implants to form a hard crust and continue until the new bone is formed to achieve solid fracture healing. A longer period is required for patients with significant bone defects to maintain the mechanical properties of the endoprosthesis. The degradation rate of the implanted scaffold should be maintained at approximately 0.2–0.5 mm per year to prevent premature or late degradation. Unfortunately, previous *in vivo* studies have demonstrated that Mg alloys exhibit a rapid degradation rate, failing to meet the requirements for bone repair. Thus, it is difficult for Mg to provide adequate mechanical support, which may lead to risks such as air pockets, hemolysis, and osteolysis. However, most observations suggest that the degradation of Mg alloys *in vivo* is slower than that measured *in vitro* (Hiromoto et al., 2015). These rapid degradation characteristics are mainly related to their electrochemical properties. The standard electrode potential of Mg is approximately −2.37 V, which is lower than Zn (−0.7 V) and Fe (−0.89 V). Due to the presence of impurities on the surface of Mg alloys, intermetallic galvanic corrosion accelerates their degradation (Ren et al., 2019). The premature degradation of Mg bone implants leads to premature loss of mechanical integrity. The degradation process is accompanied by the continuous release of H gas, which causes an elevated pH and the appearance of minor bubbles in the implanted portion. The degradation behavior of Mg and its alloys is mainly electrochemical, and its corrosion behavior involves the anodic dissolution and cathodic reduction of Mg alloys with the following equations (Xin et al., 2011):

$$Anodic\;reaction:\;Mg\;\to \;{Mg}^{2+}+2e$$ (1)

$$Cathodic\;reaction:\;2H_{2}O+2e\;\to \;H_{2}+2{OH}^{-}$$ (2)

$$General\;reaction:\;Mg+2H_{2}O\;\to \;Mg{\left(OH\right)}_{2}+H_{2}$$ (3)

$$Dissolution\;reaction:\;Mg{\left(OH\right)}_{2}\;\to \;{Mg}^{2+}+2{OH}^{-}$$ (4)

Magnesium can easily interact with electrolytes in the body’s fluid environment (Eq. 1) and oxidize to cations, and the released electrons are used in the cathodic reduction reactions. Hydrogen ions generally gain electrons to form H gas (Eq. 2). Hydrogen precipitation is the primary cathodic reaction for Mg because its electrode potential is considerably lower than the equilibrium electrode potential for the H reaction. The Mg matrix typically suffers from severe anode dissolution owing to non-equilibrium grain boundaries caused by the poor corrosion resistance of Mg alloys (Yan et al., 2021). Therefore, the overall Mg corrosion reaction produces Mg(OH)_2_ and H_2_ (Eq. 3). Additionally, galvanic coupling corrosion can occur at the junction because of the potential difference between the Mg matrix and grain boundaries in the alloy. After corrosion, a heavy Mg(OH)_2_ layer is usually formed on the Mg matrix (Eq. 3). Based on the reactions described above, the corrosion product layer on the Mg matrix consists of an inner thick MgO layer and an outer heavy Mg(OH)_2_ layer (Li et al., 2008). When this metal oxide covers the Mg alloy surface, it acts as a kinetic barrier, temporarily preventing further ion or chemical reaction migration across the metal surface, which has low solubility in water and protects the Mg substrate (Hiromoto et al., 2015). However, the internal Mg oxide layer is insufficiently dense for providing sufficient corrosion protection because the ratio of the essential unit volume of Mg oxide to the molar volume of Mg metal, the Pilling-Bedworth ratio, is 0.8–1, preventing complete coverage of the oxide on its surface (Peng et al., 2016). Additionally, Mg alloys produce a large amount of H while generating the film (Eq. 3). In releasing H outward, the surface film of the Mg alloy is impacted, which in turn leads to the destruction of the surface film and loss of protection. Simultaneously, the high concentration of Cl^−^ in the body fluid usually destroys the Mg(OH)_2_ protective layer and causes pitting. Consequently, Mg and its alloys generally exhibit an excessively rapid corrosion rate in physiological environments.

### 3.2 Typical strategies to improve the corrosion resistance of Mg alloys

Numerous researchers have devoted extensive efforts to modulating the *in vitro* and *in vivo* AM Mg alloy degradation rate to address the long-term safety of porous scaffolds, accomplishing inspiring progress (Wang and Shi, 2011; Dutta et al., 2020; Nasr Azadani et al., 2022). Purification and alloying are two strategies used to address the rapid degradation of AM-Mg alloys. In addition to these two methods, other approaches can further improve the corrosion resistance of AM-Mg-based implants. Surface treatment has been an effective strategy for regulating degradation rates in recent years (Li and Zheng, 2013). Moreover, the preparation of Mg metal matrix composites (MMC) by adding reinforcing particles is another novel method to improve the degradation resistance (Dezfuli et al., 2017a; Dezfuli et al., 2017b; Kumar et al., 2019; Nie et al., 2022). However, these two measures for improving the corrosion performance of AM-Mg are currently less studied. More experimental designs are needed in the future to confirm their anti-degradation properties and impact on biocompatibility. In summary, four typical methods have been proposed for controlling the degradation rate of Mg alloys, namely, purification, alloying treatments, surface coating, and MMCs. These methods are described in detail below.

#### 3.2.1 Purification

There are two primary types of Mg corrosion, namely, self-corrosion and galvanic corrosion. Minimizing impurities and the secondary phase in the Mg matrix can effectively limit the occurrence of galvanic corrosion (Atrens et al., 2018). Based on this phenomenon, researchers have utilized a purification approach to enhance the corrosion resistance of Mg alloys (Yu et al., 2018). Impurities mainly include Fe, Ni, and Cu, as their tolerance limits in the Mg matrix are minimal (Fe, Ni < 0.005 wt%, Cu < 0.05 wt%) (Han et al., 2015). Ni and Cu tend to form Mg_2_Ni/MgCu_2_ compounds with Mg and distribute around the grain boundaries; the high potential difference that forms accelerates the self-corrosion of Mg (Cao et al., 2013). When the content of these impurities does not exceed the permissible limits, the corrosion rate of Mg is low. However, when the impurity content exceeds the allowable limit, the corrosion rate sharply increases. Some studies demonstrated that when the Fe content is reduced to <26 ppm, the corrosion rate of the Mg matrix decreases from 14.9 to 0.5 mm/year (Clover et al., 2005; Qiao et al., 2012). Improving the purity of Mg alloys is mainly achieved by using metallurgical methods, but generally at a higher economic cost. To reduce the impurity content, it is necessary to strictly control the laser process-related parameters. The optimal forming process parameters must be matched to the surface state of the powder to destroy the surface oxide layer without introducing porosity defects (Liu et al., 2019b). Another effective method is to produce “harmless impurities,” which is similar to the alloying method, by converting impurities into harmless substances that do not affect the corrosion of Mg alloys, that is, by adding elements that easily react with impurities. Prasad et al. (2012) incorporated an appropriate amount of Zr into Mg-X binary alloys (X = Y, Si, Sn, Ca, Sr, Ce, Gd, Nd, La, Mn, or Zn), and found that Zr reduced the corrosion rate by reacting with Fe impurities to form an Fe_2_Zr precipitation phase. Additionally, Zn additions can effectively reduce Fe and Ni impurity elements, thereby reducing the local corrosion of the Mg alloy (Wang et al., 2021a). Manganese by itself is detrimental to Mg alloys because Mn is very cathodic, and the presence of Mn causes micro-electro-couple corrosion with the Mg crystal phase. However, Mn can inhibit the presence of other impurity elements in Mg alloys, thus improving their corrosion resistance. Manganese can generate intermetallic compounds with Fe impurities in Mg alloys and precipitate out, reducing the content of impurities in Mg alloys (Zhang et al., 2018a).

#### 3.2.2 Alloying

Another viable approach is to employ alloying elements to reduce the volume and size of the secondary phase in the alloy matrix, thereby reducing galvanic corrosion in the α-Mg matrix caused by the secondary phase. Alloying elements, such as Al, Mn, Ca, Zn, and rare earth (RE) elements, improve the corrosion potential of the Mg matrix from a thermodynamic perspective, thereby improving the corrosion resistance of Mg-based biomaterials for bone implants (Agarwal et al., 2016). Furthermore, adding other alloying elements, such as the toughening element Zn, can be considered to improve the comprehensive performance of the Mg substrate (Wang et al., 2013). It has been demonstrated that adding trace amounts of Na to the Mg-10Sn-3Al-1Zn alloy imparts a significant age-hardening effect (Elsayed et al., 2013). investigated the corrosion behavior of a Mg-Sn alloy fabricated using L-PBF. The metal corrosion resistance decreased when the Sn addition exceeded the threshold content of Sn. This phenomenon was mainly due to the slowing effect of crystal grain refinement on the corrosion rate instead of the accelerating effect of coupled galvanic corrosion caused by the secondary phase on the corrosion rate. The addition of up to 5 wt% Sn improved the corrosion performance. The grain size decreased with an increasing Sn content, whereas the new Mg_2_Sn phase increased.

RE elements can improve the mechanical strength and creep resistance (Cui et al., 2020), refine the grain, and synthesize precipitated phases. RE elements, such as Y, lead to the formation of a protective antioxidant film on the surface of the Mg matrix after heat laser fabrication; WE43 alloys demonstrate excellent degradation resistance (Liu et al., 2022a). The oxidation activities of the RE elements of Y, Nd, and Gd are higher than that of Mg. Therefore, Y, Nd, and Gd are preferentially oxidized, as compared to the inner Mg matrix, forming a solid oxide film on the surface of the Mg matrix. This oxide film increases the ignition point of the powder material and reduces the risk of ignition and explosion during operation. Additionally, after alloying Mg with Ca and Y, a more stable hydroxide layer with a lower chemical reactivity is formed on the surface of the Mg matrix, making the alloys more stable in simulated body fluid (Velikokhatnyi and Kumta, 2010). From a material perspective, single-phase Mg-RE alloys can avoid galvanic corrosion. RE elements can concurrently improve the mechanical properties of Mg alloys and increase corrosion resistance by strengthening the solid solution (Chou et al., 2013).

#### 3.2.3 Surface modification of Mg alloys

Surface modification affects the biodegradation rate of AM Mg alloys (Matras, 2019). In response to the fast degradation rate, local alkalinization, and local gas emission of Mg alloys, a wide variety of surface modification approaches have been introduced to construct a corrosion barrier layer on the surface of the Mg alloy body to inhibit degradation (Guo et al., 2022). Standard methods include physical surface coatings, surface structure, chemical modification, and a combination of these approaches (You et al., 2021). Different physical layer materials, including bioactive ceramics, polymers, chemical conversion coatings, metal plating, and anodic oxide films, have been designed to modify the corrosion performance of Mg alloys (Chandra and Ram Chauhan, 2021). Because a large amount of physical polymer coating forms on the Mg substrate through physical interaction *via* immersion or dipping, the layer tends to easily detach from the substrate when inserted into the bone (Li et al., 2022). Chemical changes synthesize new metallic phases on the surfaces of Mg alloys through chemical or electrochemical reactions to inhibit the corrosion process. This method removes the natural oxide layer, which has poor passivation properties. Chemical modifications typically include acid etching, alkali heat treatment, fluoride treatment, anodic oxidation, and micro-arc oxidation (MAO) methods. As the struts of PBF specimens usually have adhering powder particles, smoothing the surface can modify the biomechanical properties of AM Mg. The polishing procedures employed thus far, including chemical etching, electrochemical etching, and sandblasting, have not achieved a uniform, smooth surface at the periphery and center of the porous supports (Li et al., 2018a). For example, it was demonstrated that the corrosion resistance of a treated WE43-Mg alloy was significantly enhanced by coating the MAOed alloy with chitosan and polystyrene layer-by-layer (Zhen et al., 2015; Jin et al., 2022).

#### 3.2.4 Magnesium-based MMCs

The corrosion rate of Mg-based MMCs can be adjusted by selecting different reinforcing particles and their amounts. Several bioactive bioceramics, including Hydroxyapatite (HA) (Shuai et al., 2017a; Hiromoto et al., 2021), Tricalcium phosphate (TCP) (Zheng et al., 2017; Narita et al., 2019; Dang et al., 2020; Pan et al., 2020), and bioglass (BG) (Zhang et al., 2016; Duan et al., 2020; Singh et al., 2020), have been utilized as reinforcement particles in Mg-based MMCs. The addition of hydroxyapatite enhances the compressive properties of AM Mg. However, the tensile properties are reduced, and Mg containing HA exhibits a lower degradation rate than pure Mg. Jo et al. (2011) fabricated an MgF_2_/HA coating on a pure Mg surface, and *in vivo* experiments demonstrated that the coating decelerated the implant degradation rate. β-TCP has low biodegradability and a composition similar to that of natural bone minerals, and its degradation products can act as a base material for bone regeneration. Additionally, the mechanical properties, plasticity, and corrosion resistance were improved with an increasing β-TCP content (Gu et al., 2019). BG ceramics exhibit good corrosion resistance and biocompatibility. The preparation of BG coatings on the surface of Mg alloys typically involves sol-gel dip coating and electrophoretic deposition methods (Yadav et al., 2020). Although BG coatings on the surface of Mg alloys prepared using the sol-gel method normally exhibit low bonding strength, the results demonstrated that the microcrystalline glass coating lost its protective effect on the Mg substrate within a relatively short period when the external compressive stress was >25 MPa (Zhang et al., 2016). L-PBF-formed MMCs have been investigated as an effective method to improve biodegradation ability. Tao et al. (2020) revealed that doping graphene oxide in ZK30 powder could effectively reduce the content of the MgZn_2_ precipitated phase in the L-PBF deposited state, thus enhancing the biodegradation resistance.

### 3.3 AM parameters that determine the corrosion resistance of AM-Mg alloys

The biodegradation of AM Mg depends on the selected material, design, and manufacturing process parameters. Laser-based AM provides unique opportunities for the preparation of interconnected porous Mg scaffolds with a high geometric freedom. The printing parameters and laser processes control the size and distribution of these micropores (Xu et al., 2021b). An ideal scaffold must have interconnected pores to facilitate the flow of body fluids and cell growth and a porous structure that is reproducible using the chosen manufacturing method. However, studies on the complex interaction between the design of pores and corrosion properties of AM-Mg alloys are still in their infancy, and a considerable amount of work is urgently needed.

The geometric design of the porous structure affects the degradation performance of AM Mg scaffolds. Wang et al. (2021b) demonstrated the effect of geometric design on the dynamic degradation performance of AM Mg scaffolds for the first time. Three central permeable systems were designed (biomimetic, diamond, and sheet-based grids) and subjected to the L-PBF process. The sheet-based gyroid structure outperformed the other structures in terms of degradation endurance and uniform degradation phase. Based on these findings, geometrical design could be a promising method for producing desirable bone substitutes for the treatment of critical-size load-bearing defects. Crystallographic texture also affects the corrosion rate. Magnesium ions dissolve less in the direction of loose crystallographic stacking. Schultze et al. (2004) found that textured AZ31 exhibited higher corrosion resistance when the crystallographic plane was the significant plane parallel to the surface. Similar results were found for extruded AZ31 samples with a preferential crystallographic orientation (Jiang et al., 2017). L-PBF models with Mg and Zn have strong textures with a preferential growth direction, which may increase the corrosion rates on the upper and lower surfaces of the L-PBF samples.

The specific surface area is probably the most critical factor affecting the corrosion rate of AM Mg alloy porous scaffolds. Metallic porous scaffolds corrode faster than bulk metals because of their larger surface area connected with the corrosive medium (Yazdimamaghani et al., 2017). Li et al. (2018a) reported that the weight loss of L-PBF porous WE43 was approximately 9% after immersion in simulated body fluid (SBF) for 7 days. However, the weight loss of the bulk WE43 plate (10 × 10 × 2 mm^3^) was approximately 5%. The geometrical features of porous scaffolds, such as porosity and surface roughness, alter the corrosion rate. Table 2 summarizes the biodegradation performance reported in recent studies on the development of L-PBF Mg-based alloys.

**TABLE 2:** Summary of the recent biodegradation performance of L-PBF Mg-based powders.

Mg alloys	Powder characteristics (μm)	Geometry and relative density	Biodegradation performance	*In vitro* test	Refs
Mg	25 (10–45)	Single track	—	—	Ng et al., (2010); Ng et al., (2011b)
Mg	25.85, 43.32 Gas atomization	Cubic; 96.13	—	—	(Hu et al., (2015)
Mg-Ca	100–200 Gas atomization	Scaffold	—	—	Liu et al., (2017)
Mg-3Zn	60 Gas atomization	Cubic; 97.9	4.6–6.7 ml/cm^2^ per day	7d SBF immersion	Chen et al., (2016)
AZ91D	59 Gas atomization	Cubic; 99.5			Wei et al., (2014)
ZK30-xCu	10	Non-porous block	Biodegradation rate: ZK30 < ZK30–0.1Cu < ZK30–0.2Cu < ZK30–0.3Cu	—	Xu et al., (2019)
Mg–5.6Zn–0.5Zr (ZK60)	30	Non-porous block	Hydrogen evolution rate in Hank’s solution: 0.006–0.019 ml/cm^−2^ h^−1^	48 h immersion in Hank’s solution	Shuai et al., (2017b)
ZK60-Nd	—	Porous scaffold cylinder	1.56 mm per year	-	Shuai et al., (2017c)
ZK60-xCu	4.5–13.6	Non-porous block	ZK60-xCu: 9.5–11.3	5d MG-63 direct contact	Shuai et al., (2018)
WE43	25–60	Porous scaffold cylinder	0.17 ml/cm^2^/d	3d MG-63 direct contact	Li et al., (2018b)
WE43	25–60	Porous scaffold cylinder; pore size: 600 μm	—	—	Li et al., (2018a)
WE43	25–60	—	7.2 mm per year	—	Zumdick et al., (2019)
WE43	21.77–64.46	Porous scaffold cylinder; pore size: 300–500 μm	0–4 h: 1 ml/cm^2^/h 4–12 h: 4.26 ml/cm^2^/h	12 h immersion in Hank’s solution; 7 d direct contact with human bone marrow mesenchymal stem cells	Liu et al., (2022c)
Mg-Nd-Zn-Zr (JDBM)	—	Porous scaffold cylinder; Pore size: 800 μm	Approximately 40% weight loss after 21 d of static immersion	21 d DMEM immersion	Wang et al., (2021b)
JDBM	63.9 ± 14.5	Pore size: 300–400 μm	Biodegradation rate: 0.039 ± 0.003 g/d	10d SBF immersion; 21 d MC3T3-E1 differentiation	Xie et al., (2022)
Zn-xMg	20	Porous scaffold cylinder; Pore size: 600 μm	Biodegradation rate: Zn < Zn–0.1 Mg < Zn–0.2 Mg < Zn–0.5Cu	28d Hank’s immersion; 5 d MC3T3-E1 direct contact	Qin et al., (2022)

Previous studies have reported that the corrosion rate of Mg alloys can be reduced by grain refinement (Sezer et al., 2018). Fine grains may result in a decreased corrosion rate in a passivated environment because of the formation of corrosion products. Several studies have demonstrated that the corrosion rates of Mg and its alloys decrease with a decreasing grain size (Marco et al., 2017; Niu et al., 2021). A reduction in grain size results in more uniform corrosion products, which facilitates the formation of a dense layer of Mg(OH)_2_ and MgO, thereby reducing the corrosion rate.

Multiple factors influence the corrosion rate of L-PBF biodegradable metal porous scaffolds. The passivated surface of the corrosion products counteracts the theoretical increase in the corrosion rate caused by grain refinement. The firm texture of the L-PBF samples results in an anisotropic corrosion rate. The rapid cooling of the L-PBF method significantly influences the morphology, quantity, and distribution of the secondary phase, and local heating and cooling can produce significant residual stress. Both of these factors affect the corrosion rate. The aforementioned L-PBF factors are considerably more sensitive to corrosion.

Overall, AM-Mg-based alloys can be fabricated using the four methods mentioned above to control their degradation rates; however, alloying, purification, and surface treatment are the most widely used methods. Moreover, more MMC-related matrix metal-based printing for AM-Mg degradation resistance control is needed because of the adjustment of the printing parameters.

## 4 Current status of the biomechanical properties of AM Mg alloys for the treatment of bone defects

One of the main challenges for bone defect implantation is that the biomechanical strength of Mg bone implants with AM porous scaffolds in critical weight-bearing areas of the body requires further improvement. Although the fabrication of AM bulk Mg for cortical bone fixation and AM porous Mg for trabecular bone replacement is desirable because of the porous nature of the printed scaffold, they reduce its elastic modulus and strength. Implanted AM Mg alloys require sturdiness without sacrificing their corrosion resistance and ductility (Chen et al., 2020). Specifically, the mechanical parameters of cortical bone include a tensile yield strength (YS) of 53 MPa (tested perpendicular to the bone axis) and 124–174 MPa (stretched parallel to the bone axis), a compressive YS of 1–20 MPa, a compressive strength of 103–140 MPa, and a modulus of elasticity of 1–35 GPa, whereas bone trabeculae exhibit a compressive YS of 2–12 MPa and a modulus of elasticity of 0.1–5 GPa (Wu et al., 2014). The mechanical properties of human bones vary from person to person and are strongly correlated with age, bone condition, bone shape, and the presence of combined bone metabolic diseases (Wu et al., 2018). Therefore, AM pure-Mg exhibits elastic modulus values (27–35 GPa) similar to those of cortical bone. However, the tensile YS of AM pure Mg (51 MPa) is slightly lower than that of cortical bone. Furthermore, bone implants should reduce stress-shielding effects while maintaining a degradation rate that matches the bone tissue healing cycle.

Biomechanical strength, including the yield and ultimate tensile strengths, is generally influenced by chemical composition, grain size, and printing parameters (Sezer et al., 2018). Similar to the factors affecting the degradation behavior of Mg alloys using L-PBF, the mechanical properties of Mg alloys formed using L-PBF are determined by several factors linked to the influence of the metal composition of the print-prepared powder material, including the powder grain size, structure, particle pore size, laser process parameters of the forming process (for example, hatch spacing and scanning speed), and other factors. Herein, the biomechanical properties of relevant AM Mg alloys are reviewed (Table 3), and the effects of various relevant factors are discussed.

**TABLE 3:** Biomechanical properties of AM biodegradable Mg.

Materials	Mechanical properties				Refs
	Dimensions	Hardness (Hv)	CS	YM	
Mg	—	60–97	—	20.8–38	Ng et al., (2010); Ng et al., (2011b)
Mg	Cubic 6 × 6 × 6 mm	44.8–52.4	—	—	Hu et al., (2015)
Mg-Ca	—	60–68	111.9	1.264	Liu et al., (2017)
Mg-3Zn	Cubic 5 × 5 × 3 mm^3^	62–72	—	—	Chen et al., (2016)
Mg-3Zn/xHA	10 × 10 × 5 mm^3^	60.5–80.3	—	—	Shuai et al., (2017a)
AZ91D	—	85–100	U/Y/E 296/254/1.83		Wei et al., (2014)
ZK30-xCu	—	80–98	—	—	Xu et al., (2019)
ZK60	Cube 6 × 6 × 6 mm^3^	70.1–89.2	131.6 MPa	—	Shuai et al., (2017b)
ZK60-Nd	Cube 8 × 8 × 8 mm^3^	85.4–124.8	138.3–183.4 MPa	—	Shuai et al., (2017c)
ZK60-xCu (x = 0, 0.2, 0.4, 0.6, and 0.8 wt%)	—	ZK60: 80.5; ZK60–0.8Cu: 105.2	ZK60–0.4Cu:158.3; ZK60-0.6Cu: 100–120; ZK60-0.8Cu: 167–211	—	Shuai et al., (2018)
WE43	—	—	27	0.76	Li et al., (2018b)
WE43	—	—		296	Zumdick et al., (2019)
WE43	Cube 6 × 6 × 6 mm^3^	72.43–77.41	Strut size: 4.37–21.21 Scaffold structure: 41.38–24.49	Strut size: 154.5–790.2	Liu et al., (2022c)
Mg-Nd-Zn-Zr	Diamond unit	—	54.80 ± 6.43	1.98 ± 0.02	Xie et al., (2022)
Zn-xMg (x = 1, 2, and 5 wt.%)	Cube 5 × 5 × 5 mm^3^	Zn: 41.2 ± 2.3; Zn-5 Mg: 200.4 ± 1.2	40.9 ± 0.4, 35.3 ± 0.7, 23.6 ± 0.4 MPa	1.17 ± 0.11, 1.34 ± 0.07, 1.02 ± 0.04	Qin et al., (2022)

CS, compressive strength (MPa); YM, Young’s modulus (GPa); U, ultimate strength (MPa); YS, yield strength (MPa); E, elongation (%).

Additionally, the static mechanical performance index evaluation and *in vivo* fatigue behavior of AM-degradable Mg alloys require further analysis. The corrosion fatigue behavior is particularly relevant to the biomechanical maintenance of AM Mg alloys because biodegradation tends to shorten their fatigue life and exacerbate the appearance of surface cracks (Li et al., 2020a). Therefore, it is essential to improve the fatigue resistance of AM-degradable Mg alloys to ensure the success of bone implants. Most studies have reported that metallic materials prepared using AM techniques exhibit poor fatigue resistance (Wang et al., 2021c). The fatigue behavior of AM alloys is influenced by the material type, forming quality, geometric design, biodegradation, post-forming treatment, and other factors. Research on improving the fatigue performance of AM Mg has mainly focused on three aspects. First, adding RE elements can effectively enhance the fatigue performance of cast AM Mg (Bian et al., 2016). Finally, surface deformation strengthening can improve the surface strength and hardness of AM Mg. By applying an external force to the casting surface, the surface undergoes plastic deformation hardening, inhibiting the formation of fatigue cracks (Zhang et al., 2015; Ji et al., 2022).

### 4.1 Effect of alloying elements on the biomechanical properties

The mechanical properties of L-PBF components can be significantly improved by mixing nanoparticles with metals (Atrens et al., 2018). The solid solution of the alloying element in the matrix has a substantial solution-strengthening effect, refining the grain to some extent. In contrast, the secondary phase of the AM-formed ZK91D alloy exhibits a precipitation strengthening effect, and the tensile YS reaches 254 MPa (Wei et al., 2014), which is better than that of cortical bone and meets the YS requirements for biodegradable metal bone fixation devices (>230 MPa) (Venezuela and Dargusch, 2019). Table 4 summarizes the effects of different metals commonly found in alloys on biomechanics, corrosion properties, and cytotoxicity. The addition of various metals can improve the biomechanics of their metals. Recently, Yang et al. (2020) demonstrated that Li and Mg in extruded Zn exerted a more effective strengthening effect than other alloying elements, with Li displaying the most effective strengthening effect in Zn, followed by Mg. Similarly, an L-PBF Fe-Mn alloy demonstrated a significantly higher YS than that of L-PBF pure Fe (Shuai et al., 2020). Recently, Xie et al. (2022) proposed an Mg-Nd-Zn-Zr-based alloy (denoted as JDBM) using an alloying approach. The alloy innovatively uses RE elements, such as Nd, as the primary alloying element and includes trace elements of Zn and Zr. The Mg-Nd binary alloy exhibits significantly enhanced mechanical properties and corrosion resistance (Willbold et al., 2017). In contrast, Zn increases the flexibility and deformation properties of Mg alloys.

**TABLE 4:** The biomechanical property impact of standard alloying elements in biodegradable Mg alloys (Agarwal et al., 2016; Kumar et al., 2022).

Alloying element	Mechanical property refinement	Pathophysiology	Toxicology
Ca	1. Grain refinement and improved corrosion resistance 2. Surface stress reduction	Calcium is mostly stored in bones and teeth, 0.919–0.993 mg/L of blood serum	Metabolic disorder
Zn	1. Reduction of H gas generation 2. Enhanced yield stress	Blood serum level 12.4–17.4 mol/L necessary for enzyme and immune system	Neurotoxic and bone growth is hampered
Mn	Enhanced corrosion resistance	Blood serum level <0.8 μg/L. Influences bone growth and cellular functions/immune system	Neurological disorder
Cu	Anti-bacterial effect		
Sr	Grain refinement and corrosion resistance	140 mg and 99% located in the human bones	Neurological disorder
Si	Increases tensile strength, elongation, and biocorrosion resistance	Good biocompatibility	Unclear
Y	Improves elasticity and forms a protective layer on the surface of the Mg matrix	Blood serum level <47 μg	Hepatotoxicity
Ag	Increases tensile strength low corrosion resilience, and anti-bacterial effect	Blood serum level 11–26 μg/L	Unclear

The fatigue behavior of AM porous metals mainly depends on the material itself. Under similar geometric designs, AM porous pure Fe and Zn exhibit higher fatigue strengths than AM porous Mg alloys (WE43) (Li et al., 2019b; Li et al., 2020b; Li et al., 2020c), mainly because of the high tensile biomechanical properties of pure Fe and Zn. Alloying is an efficient method for enhancing the mechanical properties of Mg alloys. Alloying elements improve the initial mechanical properties of biodegradable Mg alloys and maintain long-term intact mechanical properties *in vivo* by enhancing the corrosion resistance of AM Mg. Despite their appropriate biocompatibility, Ca, Sr, and Si contribute minimally to the mechanical properties of AM Mg because of their low solubility in Mg alloys, as compared to Zn. The second phase is typically thick and dispersed along the grain boundaries. These characteristics do not affect the mechanical properties of Mg alloys. Different alloying and layering strategies can significantly improve the biomechanical properties of Mg alloys.

### 4.2 Effect of powder fabrication and L-PBF processing on the biomechanical properties

#### 4.2.1 Impact of powder fabrication on the biomechanical properties of AM-Mg implants

**FIGURE 5:**
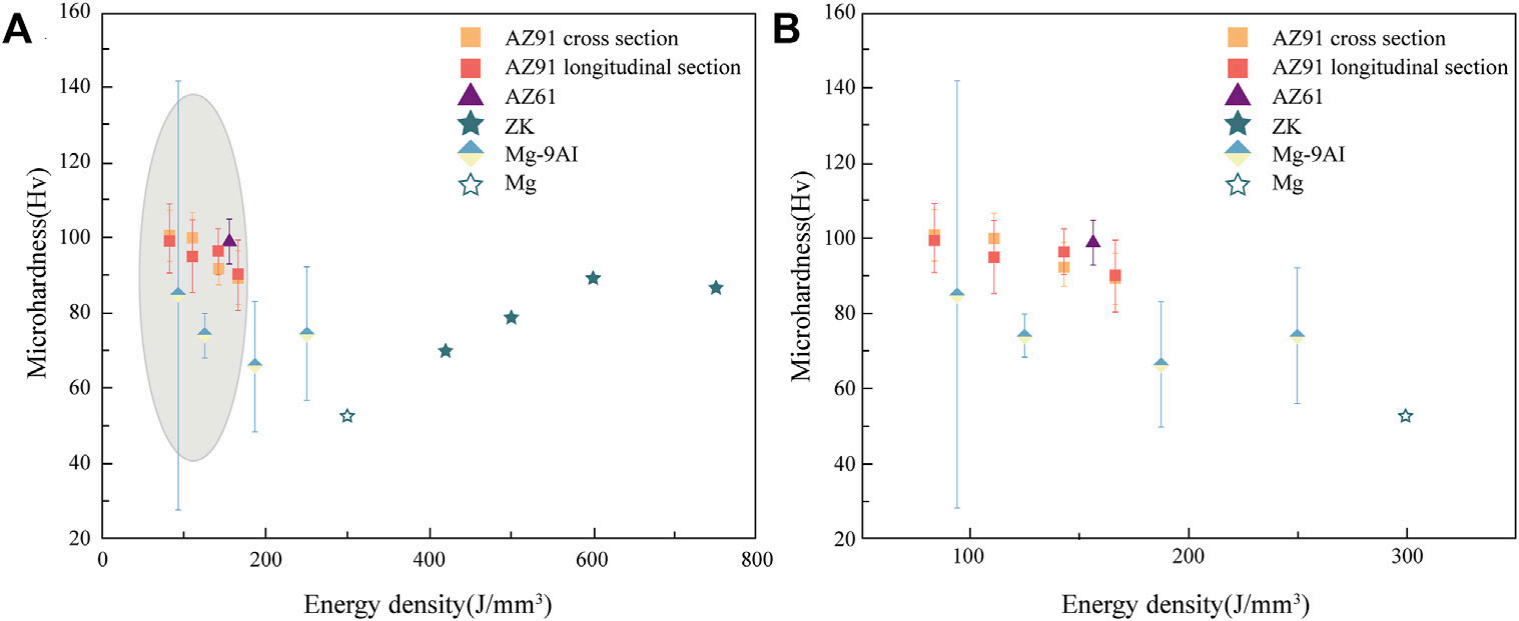
The effect of energy density on the microhardness of L-PBF Mg alloys (Liu and Guo, 2020) **(A)** Effect of energy density on the microhardness **(B)** Enlarged image of the grey area in the effect of energy density on the microhardness of L-PBF Mg alloys.

The existing results indicate that the hardness and tensile strength of the biodegradable metallic block material formed using L-PBF are higher than those prepared using conventional methods (Qin et al., 2019). However, the elongation is lower than that of rolled and extruded samples. The high density can explain the improvement in the mechanical strength and grain refinement of the L-PBF specimens; therefore, the material strength substantially increases according to the Hall-Petch slope equation (Guan et al., 2019). Li et al. (2018b) investigated the biomechanical effects of the different crystal structures of WE43 alloy porous scaffolds fabricated using L-PBF and found that porous WE43 scaffolds with diamond structural units provided adequate mechanical support with mechanical properties comparable to those of bone trabeculae even after 4 weeks of immersion in SBF. Shuai et al. (2017b) reported that the grain sizes of L-PBF-formed Mg alloy samples were 2, 4, 6, and 8 μm at the Ev values of 420, 500, 600, and 750 J/mm^3^, respectively. The cooling rate during L-PBF could reach 10^5^–10^6^ K/s, as compared to 1–10^2^ K/s in the conventional casting process, which made the grain size formed using the L-PBF technique finer than those formed using traditional preparation methods. The elastic modulus of L-PBF Mg alloys is influenced by the thickness and properties of the powder and laser energy density. The laser energy affects the microhardness of the L-PBF Mg alloy by influencing the grain size. The curves in Figure 5 illustrate the effect of the laser energy on the microhardness of L-PBF alloys, where an increase in the energy density results in a significant increase in the grain size and, ultimately, in a decrease in microhardness, which enables the L-PBF technique to yield finer grain sizes than the classical process (Pawlak et al., 2017).

#### 4.2.2 Influence of L-PBF printing process parameters on the biomechanical properties of AM-Mg implants

The biomechanical properties of AM-Mg alloys are heavily influenced by the specific PBF process employed. Internal pores, inclusions, and cracks within the struts can impair the mechanical properties of AM-Mg. The most common method is to modify process-related parameters such as the laser power, scanning speed, and hatch spacing. The effects of hatch spacing and scanning speed on the densification of an AZ61 Mg alloy were investigated, and a reduction in the hatch spacing from 0.1 to 0.06 mm resulted in a relative density of 99.4% at a constant speed of 400 mm/s and 150 W laser power. However, further reducing the scanning speed (to 350 mm/s) resulted in a decrease in the relative density, which was attributed to the residual thermal stresses generated in the sample, which resulted in the formation of microcracks. Consequently, it was determined that hatch spacing and scanning speed adversely affect the hole size and shape, respectively (Yan et al., 2021]. Ng et al. (2011a) investigated the effects of different pulsed and continuous wave laser powers on the microstructure of specimens, demonstrating that reduced power contributed to grain refinement. The grain size of the samples shaped with a continuous wave was significantly smaller than that of a pulsed wave for a similar average energy density.

Furthermore, owing to the maximum heat flow gradient perpendicular to the direction of the substrate, the specimen tissue generated anisotropy (Song et al., 1980-2015), affecting the mechanical properties of the material, thus increasing the quality of the L-PBF Mg alloy along the forming direction. Qin [18] refined the degradation properties of Mg alloys by optimizing the energy density and scanning strategies of porous WE43 for L-PBF fabrication, thereby inhibiting the *in vivo* degradation rate.

Heat treatment is an excellent method for improving the mechanical properties of AM Mg alloys. Residual stresses are generated during the L-PBF process, where the parts are stacked layer-by-layer which continuously heats and cools the metal (Shuai et al., 2017b). Optimized heat treatment after AM can effectively reduce defects and residual thermal stresses, improve the microstructure of porous materials, and eventually enhance the biomechanical features of the lattice structures (Zhang et al., 2018b). The surface properties of the pillars that make up the lattice structure can also be further optimized by surface treatments, such as sandblasting and chemical etching, which allow for a smoother surface on the AM porous structure while removing unmelted powder particles. Therefore, heating and surface treatment may be required to further enhance the biomechanical properties of AM porous implants before testing them *in vivo* (Ahmadi et al., 2019).

### 4.3 Other research advances for enhancing the mechanical properties of AM-Mg alloys

#### 4.3.1 Application of finite element analysis to the biomechanics and degradation of AM biodegradable Mg alloy bone implants

Owing to its low cost and high efficiency, as compared to conventional investigation methods, FEA is gaining popularity for evaluating the biomechanical properties and simulating the deformation and degradation of Mg alloys (Chen et al., 2018). FEA has emerged as a practical tool to predict the biomechanical behavior of the bone-implant interface and to identify areas of concentrated stress loading (Campbell and Glüer, 2017; Liu et al., 2022b). This enables the load distribution and deformation to be optimized under different loading conditions for AM fabrication (Yao et al., 2021). Jiang et al. (2023) fabricated an auxiliary scaffold with a “Bucklicrystal” structure using AM. Furthermore, they verified its stress distribution using FEA, demonstrating its excellent potential to restore the normal physiological functions of the intervertebral disc. Huang et al. (2020) employed Mg screws as fixation devices in a goat femoral neck fracture model. They identified the possibility of evaluating the stress-loading ability at the weight-bearing sites using FEA methods. Additionally, FEA can optimize the matching between the biomechanical properties and implant load-bearing capacity. Zhang et al. (2021b) employed FEA to simulate the stress distribution of L-PBF Mg-alloy implants in the alveolar bone. The results demonstrated that the stress concentration area of dental implants was not in the porous structure, and the weak part of the mechanical properties met the operational requirements. Wieding et al. (2013) applied FEA methods to evaluate Ti alloy scaffolds to treat sizeable segmental bone defects, further adjust their porosity and material quantity, and optimize the Ti alloy material structure. Therefore, performance forecasting can be accurately calculated for AM Mg products before clinical operations. More efficient digital design solutions can be refined and constructed based on actual clinical needs.

**FIGURE 6:**
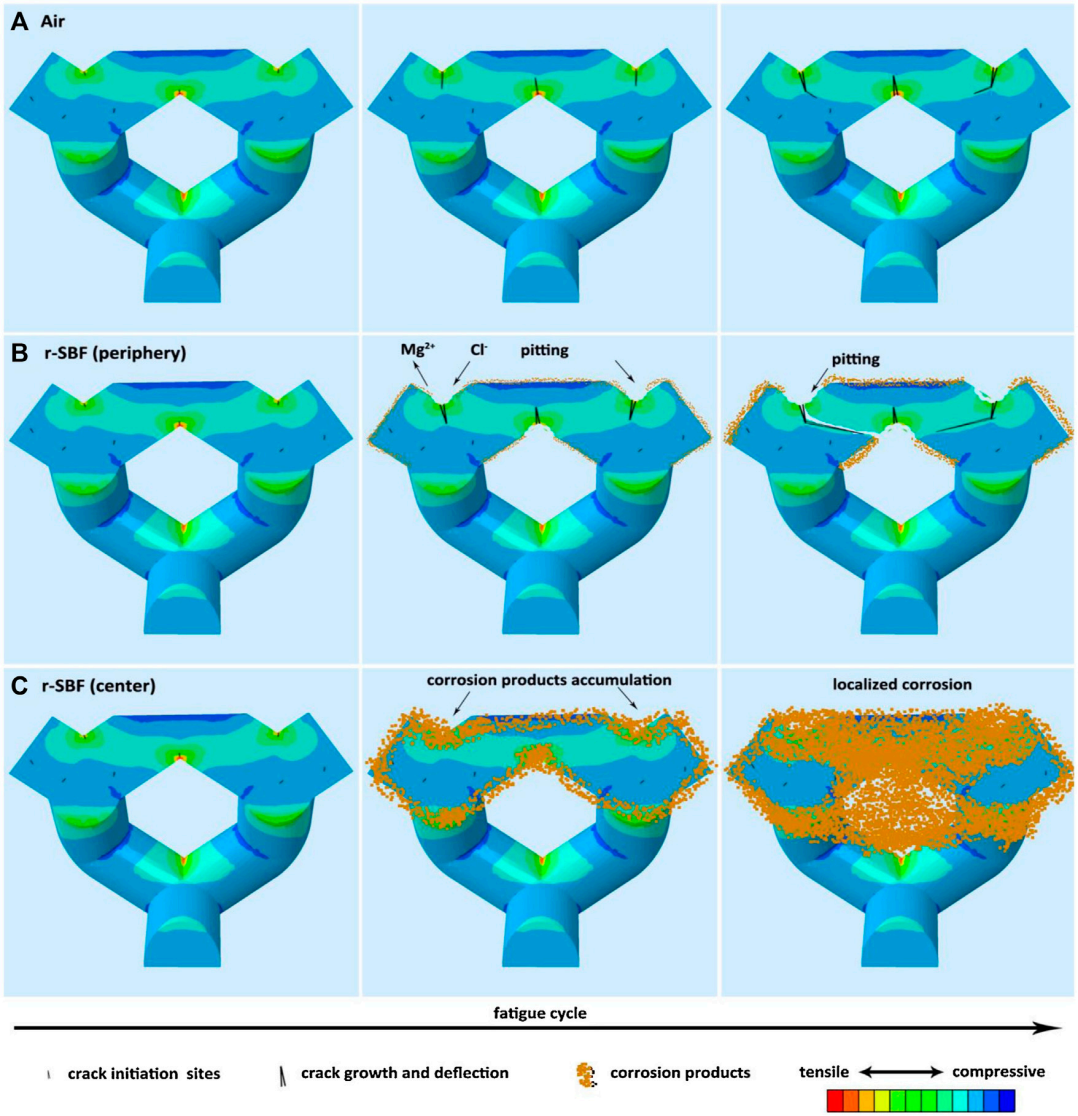
Schematic illustration of the fatigue mechanism of AM Mg alloy scaffolds (Li et al., 2019b). **(A)** In air, **(B)** in r-SBF on the periphery, and **(C)** in r-SBF at the center.

Simulated fatigue resistance analysis using FEA can effectively reduce the number of standard tests and avoid wasting labor and materials. Moreover, the FEA simulations exhibit clear patterns under different compressive strengths, reflecting its predictive value for optimizing the dynamic fatigue testing performance of products. Figure 6 shows that the FEA method simulated the fatigue and biodegradation patterns of the WE43 alloy in different immersion solutions. It was demonstrated that the biodegradation distribution of AM-Mg alloys was location-dependent, with more localized biodegradation prevailing at the center than at the periphery (Li et al., 2019b).

#### 4.3.2 The need for robust pre-clinical bone defect animal *in vivo* tests to evaluate the biomechanical and corrosion properties of AM Mg alloy

**FIGURE 7:**
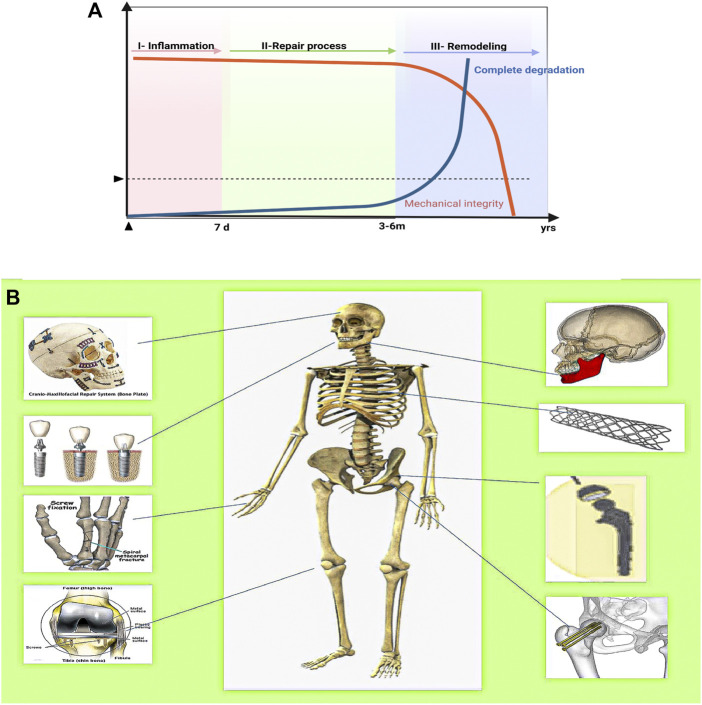
The ideal biomechanical strength curve, bone healing process, and Mg alloys for the repair of bone defects in different areas of the human body. **(A)** The ideal compromise curve between the mechanical integrity of the biodegradable metal implant, biodegradation rate, and fracture healing process. **(B)** Application of Mg alloy bioimplants in humans depending on the area affected by the disease/accident.

AM Mg implants must have an adequate initial mechanical strength to firmly support the fractured bone, at least during the early healing phase (including the inflammatory and soft callus phases), to ensure that post-surgery degradation of the fixation does not create a risk of secondary fractures (Figure 7) (Chaya et al., 2015). In 2013, Magmaris pressurized Mg alloy screws (Syntellix, Germany, Mg-Y-RE-Zr alloy) became the first Mg intraosseous implants. This was a milestone in the clinical translation of biodegradable Mg implants (Windhagen et al., 2013). Figure 7B illustrates the schematic protocol of the Mg alloy for bone defect implantation in different regions where different parts have different biomechanical requirements. However, a significant problem remains in that most current animal experiments with Mg-based implants cater for small- and medium-sized skeletal defects instead for the mid-shaft repair of long weight-bearing bones. Instead, clinical complications and the urgent need for innovative treatments mainly lie in long weight-bearing bones, such as fractures of the femoral and tibial shafts (Sun et al., 2021). Considering the relatively short period of use of L-PBF for the fabrication of biodegradable porous metals for orthopedic research, only a few *in vivo* animal studies have been conducted to date using only a few metals, predominantly inert Ti alloys. Table 5 summarizes the reported results of *in vivo* implantation, *in vivo* biodegradation behavior, and subsequent effects of L-PBF porous metals *in vivo* animal studies (Li et al., 2020a).

**TABLE 5:** Summary of the application of AM using L-PBF for orthopedic applications.Summary of the application of AM using L-PBF for orthopedic applications.

Materials	*In vivo* implantation using L-PBF				Refs
	Animal model and fracture site	Follow-up	Biodegradation	Effect	
Ti–6Al–4V	Rabbit (NZW) femurs	12 weeks	—	Promote osteogenic effect	Liang et al., (2022)
Fe-30Mn	Rabbit (NZW) femoral condyles defect	48 weeks	Maintained the structural integrity after 48 weeks	Promote osseointegration performance	Nie et al., (2021)
Ti-10Ta-2Nb-2Zr	Rabbit (NZW) femoral condyles	12 weeks	—	Promote osseointegration performance	Luo et al., (2021)
WE43	Rabbit (NZW) femurs	12 weeks	The scaffolds collapsed due to degradation after 4 weeks	Promote osteogenic effect	Liu et al., (2022c)
Mg-Nd-Zn-Zr	Rabbit (NZW) femurs	4 weeks	Maintained the structural integrity after 4 weeks	Promote osteogenic effect Anti-bacterial effect	Xie et al., (2022)
Zn-1Mg	Rabbit (NZW) femurs	12 weeks	Maintained the structural integrity after 4 weeks	Promote osteogenic effect	Qin et al., (2022)
Zn	Rabbit (NZW) femurs	24 weeks	Maintained the structural integrity after 24 weeks	Promote osteogenic effect	Xia et al., (2023b)

## 5 Outlook of AM Mg alloys for the clinical treatment of bone defects

**FIGURE 8:**
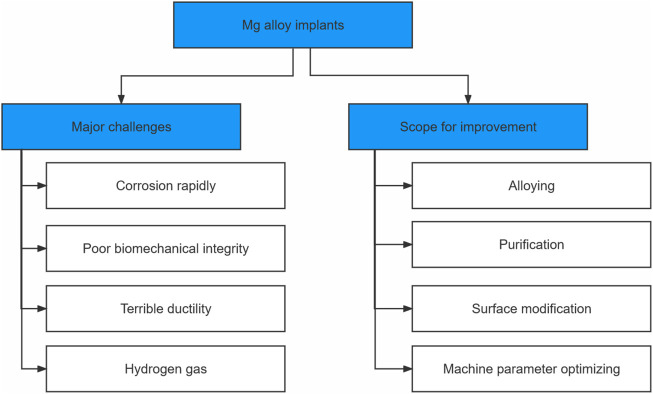
Flowchart showing the challenges and scope for improvement of Mg-alloy implants.

The combination of two primary techniques, AM and degradable Mg alloys, offers novel viable options for treating patients with bone defect implants but poses new challenges. More factors need to be considered to achieve the degradation of Mg alloys and improved mechanical properties. Significant work is still required to combine the controlled degradation of Mg alloys with the AM technique using L-PBF to leverage the strengths of each technique. This review presented the research progress and challenges of AM-Mg alloys and provided a strategy to control the degradation rate of Mg alloys and its effect on the mechanical properties. Additionally, this review discussed the role of L-PBF for the fabrication of AM-Mg alloys and the modulation of the parameters, such as the printing and powder parameters, which do not exist in isolation but affect each other, to regulate the mechanical properties of Mg alloys (Figure 8). Then, the current trends in research on AM-Mg alloys were discussed. More comprehensive evaluations of the mechanical properties of Mg alloys, especially the fatigue properties, are required. Additionally, finite element and machine learning algorithms can assist in better design and optimize structures. These studies require further *in vivo* testing of AM-Mg to better guide clinical practice.

### 5.1 Future research on controlling the degradation rate in AM-Mg with combined methods

Based on the above discussion, four typical schemes to control the degradation rate of Mg alloys, which are of great significance for improving the degradation and mechanical properties of Mg alloys, are presented. The relationship between the degradation and mechanical properties of Mg alloys is complementary. The degradation and toughening of Mg alloys during the alloying and building of Mg-based metal strategies should be minimized. On the one hand, it can concluded that the current four approaches for improving the corrosion resistance focus primarily on reducing the potential degradation of Mg alloys and achieved some success. However, their suitability for L-PBF-processed porous Mg alloys requires further *in vivo* and *ex vivo* experimental verification.

In contrast, most of the current studies involve single-method improvements. More research is needed to demonstrate the effects of multiple approaches on the degradation of Mg alloys and the improvement of their mechanical properties. Theoretically, combining multiple approaches provides a broader approach for controlling the refinement of the Mg alloy degradation and maintaining their mechanical properties (Figure 8).

### 5.2 Requirement for dedicated L-PBF-printed Mg alloy powders

From the above discussion, L-PBF can be used to obtain Mg components with a fine and homogeneous microstructure. These components are expected to exhibit better biomechanical properties and corrosion behavior due to the modulation of the parameters of the AM methods, such as L-PBF processing and powder preparation. However, dedicated Mg alloy printing powders for L-PBF processes are scarce. Hybrid materials integrate the advantages of multiple materials and offer great potential for improving the performance of bone implant materials (Gao et al., 2019a). Previous studies have demonstrated that Mg-based MMCs exhibit better corrosion behavior and biomechanical properties. Recent studies have demonstrated that LAM techniques can process MMC materials (Deng et al., 2018; Gao et al., 2019b). To date, few studies have investigated the LAM-fabricated performance of Mg-based MMCs. Therefore, the LAM of Mg-based MMCs with high performance is a new area for future research.

### 5.3 The need for more extensive evaluation and testing of the mechanical properties of AM-Mg alloys

This review shows that the current mechanical tests for AM-Mg alloys mainly focus on static parameters and less on fatigue and creep tests. The introduction of algorithms, such as finite elements and machine learning, can help better to evaluate the static and mechanical fatigue properties of AM-Mg while saving time and components, which is of great importance for the application of finished AM-Mg products to patients. Because degradable metal implants need to be evaluated against many performance indicators over an extended evaluation period, the efficiency and cost of evaluation through experimentation are urgently needed. Meanwhile, *in vivo* animal test data for AM porous-structured degradable metals is scarce, and the existing *in vitro* electrochemical and *in vitro* immersion tests cannot accurately reflect the degradation behavior of implants in the biological environment. Therefore, more animal implantation validation is still required to evaluate the degradation and mechanical properties of AM-Mg alloys and to achieve timely and personalized degradation of Mg alloys to match the needs of the clinical treatment of bone defects.

## Data Availability

The original contributions presented in the study are included in the article/Supplementary Material, further inquiries can be directed to the corresponding authors.
